# Current allele distribution of the human longevity gene *APOE* in Europe can mainly be explained by ancient admixture

**DOI:** 10.1111/acel.13819

**Published:** 2023-03-23

**Authors:** Daniel Kolbe, Nicolas A. da Silva, Janina Dose, Guillermo G. Torres, Amke Caliebe, Ben Krause‐Kyora, Almut Nebel

**Affiliations:** ^1^ Institute of Clinical Molecular Biology, Kiel University, University Hospital Schleswig‐Holstein Kiel 24105 Germany; ^2^ Institute of Medical Informatics and Statistics, Kiel University, University Hospital Schleswig‐Holstein Kiel 24105 Germany

**Keywords:** age‐related disease, aging, Alzheimer's disease, ancient DNA, ancient population genomics, evolution, longevity

## Abstract

Variation in apolipoprotein E (*APOE*) has been shown to have the strongest genetic effect on human longevity. The aim of this study was to unravel the evolutionary history of the three major *APOE* alleles in Europe by analysing ancient samples up to 12,000 years old. We detected significant allele frequency shifts between populations and over time. Our analyses indicated that selection led to large frequency differences between the earliest European populations (i.e., hunter‐gatherers vs. first farmers), possibly due to changes in diet/lifestyle. In contrast, the allele distributions in populations from ~4000 BCE onward can mainly be explained by admixture, suggesting that it also played an important role in shaping current *APOE* variation. In any case, the resulting allele frequencies strongly influence the predisposition for longevity today, likely as a consequence of past adaptations and demographic processes.

## Graphical abstract

We investigated the evolutionary history of the three major *APOE* alleles in Europe by analysing ancient DNA samples up to 12,000 years old. Our analyses indicated that selection led to large frequency differences between the earliest European populations (i.e., hunter‐gatherers vs. early farmers), possibly due to changes in diet/lifestyle. However, the allele distributions in populations from ~4000 BCE onward can mainly be explained by admixture, suggesting that it also played an important role in shaping current *APOE* variation.

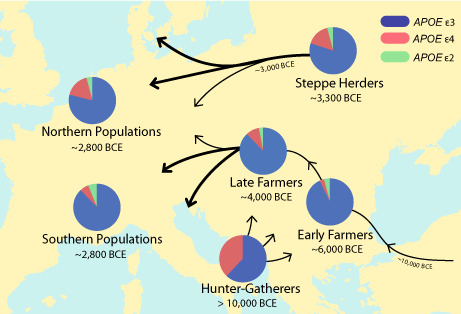

## 1 INTRODUCTION

Apolipoprotein E (*APOE*) was the first locus shown to be involved in human longevity and is still the genetic factor with the strongest effect on the phenotype (Deelen et al., 2019; Nebel et al., 2011). Two coding polymorphisms (rs429358‐T/C and rs7412‐C/T) define the three haplotypes *ε2*, *ε3*, and *ε4* which are commonly referred to as alleles. These alleles have a large influence on the development of age‐related neurodegenerative and cardiovascular disorders. For both diseases, *ε4* is deleterious compared with *ε3* (Mahley, 2016). It is responsible for most of the genetic susceptibility to sporadic late‐onset Alzheimer's disease (LOAD) (Cruts & van Broeckhoven, 1998). The detrimental effect of *ε4* is reflected in a low odds ratio of becoming long‐lived (OR = 0.60 and OR = 0.52 for 90th and 99th percentile survivors, respectively) (Deelen et al., 2019). In contrast, *ε2* is associated with a reduced risk of LOAD and is deemed a pro‐longevity allele (OR = 1.28 and OR = 1.47 for 90th and 99th percentile survivors, respectively) (Deelen et al., 2019). In addition, *ε2* has recently been reported to increase lifespan irrespective of AD (Shinohara et al., 2020).

Although *ε4* has been described as the ancestral allele (Fullerton et al., 2000), *ε3* is dominant in most parts of the world. Globally, an unequal allele distribution has been observed, especially for *ε2* and *ε4* (Singh et al., 2006). Even in Europe, frequency ranges are wide (roughly 0.74–0.88 for *ε3,* 0.06–0.22 for *ε4* and 0.04–0.12 for *ε2*), and there is a decreasing north‐to‐south cline for *ε4* (Lucotte et al., 1997). The differences in allele frequencies, both at the European and global level, have so far mainly been attributed to natural selection (Eisenberg et al., 2010; Finch, 2010; Huebbe et al., 2015, 2011; Singh et al., 2006). However, previous analyses on the evolutionary history of *APOE* considered only modern‐day sequences or genotypes (Eisenberg et al., 2010; Finch, 2010; Fullerton et al., 2000). Therefore, conclusions about the place and time of origin of the three alleles, their later dispersal or the possible influence of demographic processes had to remain vague.

Thanks to the recent advances in the field of ancient DNA (aDNA) analysis, a more detailed picture of the *APOE* history can now be drawn by incorporating allele calls from archaeologically well‐dated and well‐defined human remains. In this study, we assembled 358 diploid *APOE* genotypes from 3521 publicly available aDNA data sets spanning more than 12,000 years. Our aim was to investigate the past events which led to the current *APOE* allele distribution in Europe. For this purpose, we took into consideration that the present‐day European gene pool was basically derived from three prehistorical populations, the western hunter‐gatherers (WHG), the Anatolian Neolithic (AN) farmers and western steppe herders (Haak et al., 2015; Mathieson et al., 2015). By analysing various ancient, medieval and modern groups of sufficient sample sizes, we obtained reliable frequency estimates and established spatiotemporal allele trajectories (Marciniak & Perry, 2017). We concluded that past admixture events played a greater role in shaping the European *APOE* variation than previously assumed.

## 2 MATERIALS AND METHODS

### 2.1 Processing ancient DNA data and calling *APOE* variants

Genomic data of Eurasian samples were acquired from a large compendium of published sources (as cited by the Allen Ancient DNA Resource (AADR), https://reich.hms.harvard.edu/allen‐ancient‐dna‐resource‐aadr‐downloadable‐genotypes‐present‐day‐and‐ancient‐dna‐data) through the European Nucleotide Archive (https://www.ebi.ac.uk/ena/browser/home). Additionally, 216 shotgun‐sequenced genomes were acquired from https://reich.hms.harvard.edu/ancient‐genome‐diversity‐project as well as whole‐genome sequencing data of 42 individuals from the Late Neolithic site Niedertiefenbach (Immel et al., 2021).

Data was preferentially obtained in BAM format and in cases where such a format was unavailable, the raw *fastq* files were used instead. The methodology used to process the samples in *fastq* format was previously described by Immel et al. (2021). To minimize downstream genotype errors, the terminal positions of reads with >0.05 deamination rates were trimmed off with bamUtil v1.0.15.

BAM files from samples that passed the quality control step were piled up using SAMtools (Li et al., 2009) v1.15, filtering by base and mapping quality >20. The *APOE* polymorphic sites (rs429358‐T/C and rs7412‐C/T) were called using bcftools (Li, 2011) v1.15, removing genotypes with a depth of coverage <3. Samples missing a diploid genotype at both polymorphic sites were removed and not considered for further analysis. We completely avoided the use of pseudo‐haploid calls, unlike a previous study (Abondio et al., 2019). This was necessary to obtain more reliable allele frequencies and to be able to distinguish between homozygous and heterozygous samples.

### 2.2 Population analysis

**FIGURE 1:**
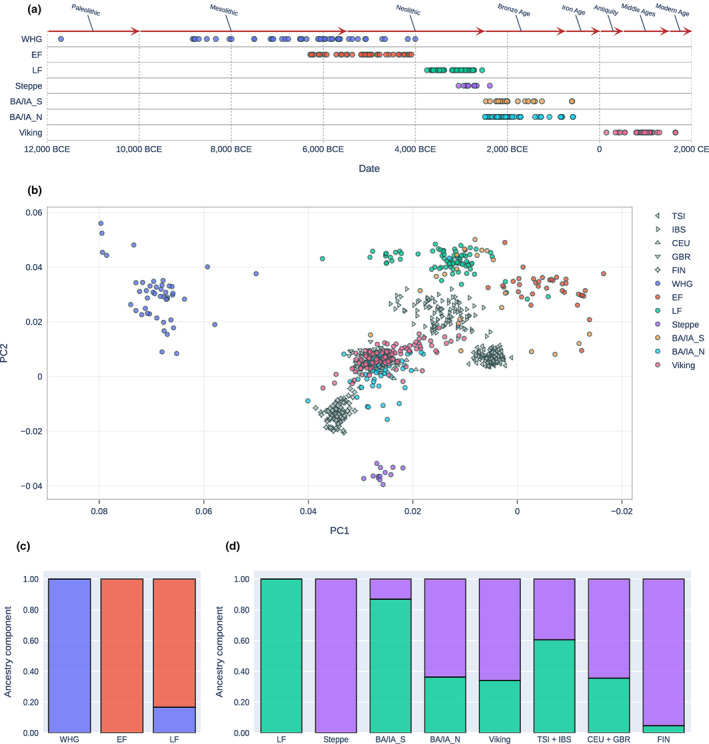
Population genetics of all samples used to create *APOE* allele frequencies. (a) Time‐points of samples. (b) Principal component analysis of ancient individuals (358 samples—colored) and 1000 Genomes populations (CEU, GBR, IBS, and TSI—gray) projected onto modern West Eurasian variation. (c, d) Supervised admixture‐analysis of ancient samples using WHG and EF (c) and LF and steppe populations (d) as parental components. BA/IA_N, Bronze Age/Iron Age North Europe; BA/IA_S, Bronze Age/Iron Age South Europe; EF, European early farmers; LF, European late farmers; Steppe, Steppe herders originating from Yamnaya region; Viking, Vikings from North Europe, Middle Ages; CEU, GBR: Northern modern European 1000 Genomes populations; TSI, IBS: Southern modern European 1000 Genomes populations; WHG, western hunter‐gatherers.

As described in a previous study (Immel et al., 2021), a principal component analysis was conducted on the merged genotyping data of ancient samples and selected modern reference populations from West Eurasia. The grouping of the samples into different ancient subpopulations (see Figure 1b) was based on this projection as well as archaeological data (taken from AADR annotation and Immel et al., 2021).

The proportions of parental populations in admixed more recent populations were calculated using supervised ADMIXTURE (Alexander et al., 2009) v1.3.0 analyses (100 bootstraps) on a pruned version of the previously mentioned genotyping data set. Only samples with genotype calls for at least one of the two *APOE* variants were included. Information on the parental samples used in the admixture analyses can be found in Tables S1a,1b.

### 2.3 Allele frequencies

The frequency for *ε3* was calculated from the counts of the rs429358‐T – rs7412‐C haplotype within a target population. To maximize sample size, frequencies for *ε4* and *ε2* were calculated from the counts of the allele‐defining variant only, namely rs429358‐C for *ε4* or rs7412‐T for *ε2* (Table S2). *APOE* allele frequencies were also calculated for the 1000 Genomes (The 1000 Genomes Project Consortium et al., 2015) populations CEU, GBR, IBS, and TSI as reference (Table S3). Significant differences between population frequencies were calculated using Fisher's exact test (Virtanen et al., 2020) (Table S4). The *p*‐values of all pair‐wise comparisons (*n* = 105) were corrected for multiple testing using a false discovery rate of 0.05 (Benjamini & Hochberg, 1995).

### 2.4 Testing for selection

A two‐sided binomial test was used to estimate whether allele frequencies in offspring populations could be explained by the admixture components of their parental populations. For an offspring population and two parental populations A and B, the expected allele frequency of the offspring population, $f_{\exp}$, was calculated as linear proportions of the admixture components, $k_{A}$ and $k_{B}$, and allele frequencies, $f_{A}$ and $f_{B}$, of parental populations:

$$f_{\exp}=\left(k_{A}\times f_{A}\right)+\left(k_{B}\times f_{B}\right)$$

Then, a binomial test (Virtanen et al., 2020) was applied to test for a significant deviation of the observed frequency in the offspring population from $f_{\exp}$ (Tables S5a,b).

To scan for signatures of selection, Tajima's D values for chromosome 19 were calculated from sequencing data of both ancient and modern populations. Only samples with a minimum chromosome 19 coverage of 70% (mean chr19 coverage was ~90%) and a mean depth of coverage of >5 in a 100‐kb window around *APOE* were used. Variants on chromosome 19 were initially filtered in the same way as the *APOE* variants (described above), additionally only keeping sites with QUAL >50. For each population, we calculated Tajima's D values using vcftools (Danecek et al., 2011) v0.1.16 in 15‐kb windows across chromosome 19 and compared the mean D value of the *TOMM40‐APOE‐APOC1* locus (three 15‐kb windows) to the distribution of all calculated D values. Using these same samples and variants, F_ST_ values were calculated with vcftools v0.1.16 for all chromosome 19 sites and between all populations (Table S6).

### 2.5 Software

Python 3.9.4 (Van Rossum & Drake, 2009) and Snakemake (Mölder et al., 2021) were used for the bioinformatics workflow. SciPy (Virtanen et al., 2020) was applied for statistical tests. Plotly 5.8 (Plotly Technologies Inc., 2015) was used to generate graphics/figures.

## 3 RESULTS

We scanned the two *APOE* allelic sites (rs429358‐T/C and rs7412‐C/T) in 3521 published aDNA samples from the past 12,000 years. Of these 3521 samples, only 10.2% (358) passed all filtering steps (see Methods). The samples were divided into various groups based on their archaeological context, such as their geographical origin and chronology (Figure 1a) as well as their genetic ancestry components (Figure 1b). Supervised admixture analysis (Figure 1c,d) was performed to trace genomic changes that had been caused by the two major demographic events in Europe during the period studied.

The first demographic event occurred around ~4000 BCE and saw the emergence of the late farmers (LF). This population formed through the admixture of local WHG with the early farmers (EF), who had migrated to Europe from Anatolia (Haak et al., 2015; Immel et al., 2021; Mathieson et al., 2015; Olalde et al., 2014) (Figure 1c). The second significant admixture event happened around 2700–2500 BCE when a large group of western steppe herders associated with the Yamnaya culture moved to Europe and mixed with the LF (Figure 1d). This last episode resulted in an unequal admixture distribution across Europe, with a higher steppe component in the north and a higher farmer component in the south, both of which can still be seen in populations today (Haak et al., 2015).

**FIGURE 2:**
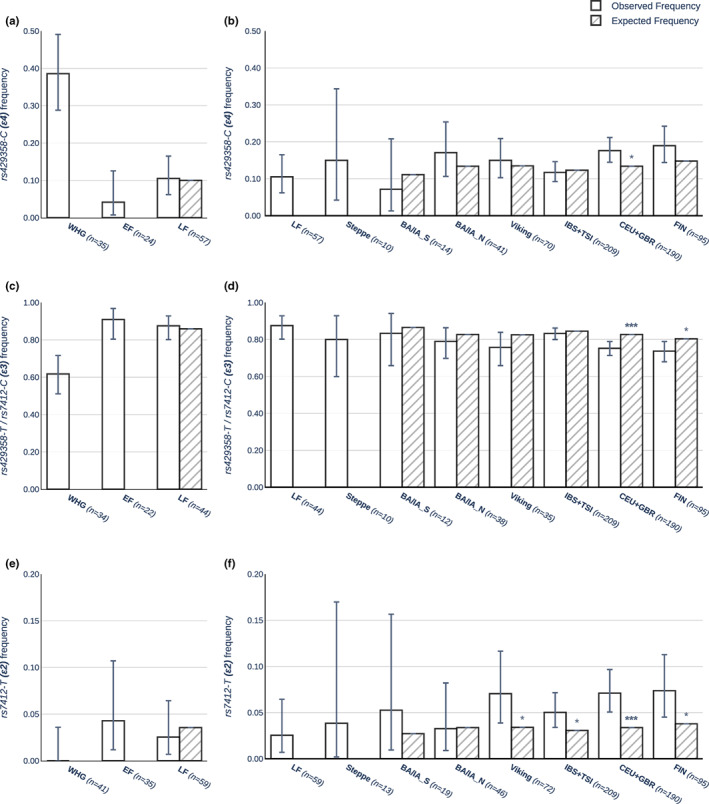
Observed (solid white) and expected (shaded) *APOE* allele frequencies of ancient populations. (a, b) *ε4* frequencies. (c, d) *ε3* frequencies. (e, f) *ε2* frequencies. Expected frequencies were calculated as a product of parental allele frequencies and parental admixture components in two demographic steps (WHG and EF form LF; LF and steppe form modern European gene pool (BA/IA_S, BA/IA_N, Viking, IBS + TSI, CEU + GBR). A binomial test was used to assess significant deviation between observed and expected frequencies (*p ≤ 0.05; **p ≤ 0.01; ***p ≤ 0.001). Error bars signify 90% confidence interval (Clopper–Pearson beta distribution) of observed allele frequencies. For population abbreviations, see Figure 1.

We noted interesting trends in the ancient *APOE* frequencies. As in modern Europeans, *ε3* was also the most common allele in all ancient groups. However, the distribution of the three *APOE* alleles differed across ancient and modern populations. This was most apparent in the earliest European groups, the WHG and the EF, who showed substantial differences in observed *APOE* allele frequencies (Figure 2) at *p* = 4.07×10^−4^ for *ε4* (Fisher's exact test, FDR‐adjusted, Figure 2a) and *p* = 0.011 for *ε3* (Fisher's exact test, FDR‐adjusted, Figure 2b). The difference in *ε2* frequency, however, remained non‐significant (*p* > 0.05, Fisher's exact test, FDR‐adjusted, Figure 2c). The WHG had a significantly higher *ε4* frequency compared with the EF and all descendant European populations, while they completely missed the *ε2* allele. On the other hand, the EF had the highest *ε3* and lowest *ε4* frequency of all examined populations, but an *ε2* frequency that was more comparable with present‐day Europeans. The admixture‐informed expected frequencies were calculated from the observed allele frequencies of parental populations in conjunction with the mixture proportions (Figure 1c,d). The *APOE* frequencies of the LF were consistent with expected frequencies, and no significant difference between observed and admixture‐informed expected frequencies was found (binomial test, *p* > 0.05). Given the LF's high EF and low WHG ancestry (0.82 vs. 0.18), it is not unexpected that LF frequencies only significantly differentiated from those in WHG (Fisher's exact test; FDR‐adjusted, *ε4: p* = 4.07×10^−4^, *ε3: p* = 0.0038, *ε2: p* > 0.05), but not from those in EF.

Following the expansion of steppe herders into Europe, *APOE* frequencies of European populations became more uniformly distributed. Although we no longer observed significant differences between any of the post‐steppe populations, we noticed interesting tendencies. Northern European groups (Bronze Age/Iron Age North, Vikings, CEU + GBR) had an *ε4* frequency comparable with that of the steppe population, which is consistent with their greater steppe component. The same observation can be made for southern European populations (Bronze Age/Iron Age South and IBS + TSI), which have a stronger LF component and, as a result, a lower *ε4* frequency. The only *ε4* frequency that significantly deviated from the admixture‐informed expected frequency was found in CEU + GBR, which was higher than expected (binomial test, *p* = 0.019). We noticed a slightly different trend for *ε2* frequencies.

While the northern Bronze/Iron Age (BA/IA_N) group had a frequency comparable with the steppe, the southern Bronze/Iron Age *ε2* frequency was higher than both LF and the steppe but did not significantly deviate from the admixture‐informed expected frequency, likely due to low sample sizes. Interestingly, the frequencies of *ε2* increased in all European common era populations and significantly differentiated from their admixture‐informed expected frequencies (binomial test; Viking: *p* = 0.031, IBS + TSI: *p* = 0.031, CEU + GBR: *p* = 3.1×10^−4^). This deviation was especially noticeable in northern populations, where *ε2* frequencies were higher than in any ancestral population. However, in this case, the power to determine accurate frequency estimates for individual populations was limited due to the low overall frequency of the *ε2* allele.

**FIGURE 3:**
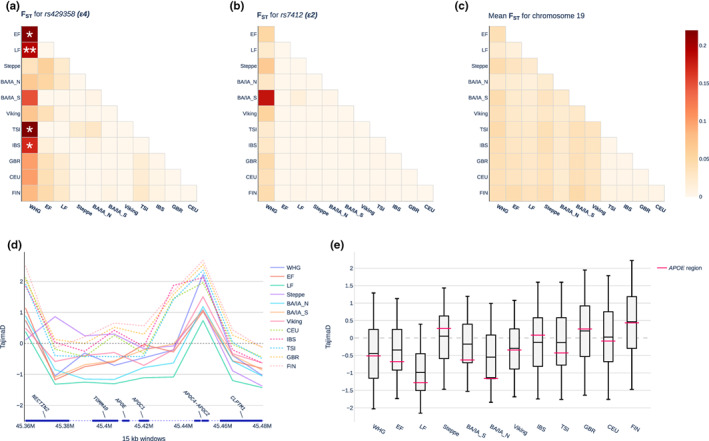
Selection scans of *APOE* region/alleles in ancient populations. (a, b). F_ST_ values for rs429358 (*ε4*) and rs7412 (*ε2*). *: upper 95th percentile of all calculated F_ST_ values on chromosome 19; **: upper 97.5th percentile of all calculated F_ST_ values on chromosome 19. (c) Mean F_ST_ values for all chromosome 19 sites that passed all filtering steps. Since the *ε3* allele is determined by both variants it has not been included as a standalone analysis. (d). Tajima's D of *TOMM40‐APOE‐APOC1* region across all ancient populations as well as European 1000 Genomes populations. (e) Tajima's D in 15‐kb windows of *TOMM40‐APOE‐APOC1* (labelled *APOE* region—red line) projected on box plots of chromosome 19 percentiles. The middle of the box represents the median, the edges of the box lower and upper quartile, and the whisker ends the 5th and 95th percentiles of all chromosome 19‐values (in 15‐kb windows). For population abbreviations, see Figure 1.

Subsequently, we assessed whether selection may have acted on *APOE* alleles. Using a selected set of high‐coverage samples from each population, we calculated cross‐population F_ST_ values for all sites on chromosome 19 as well as Tajima's D in 15‐kb windows across chromosome 19 (on which *APOE* is located) (Figure 3). We found that the F_ST_ for the two *APOE* allelic sites was highest between WHG and other populations (Figure 3a,b,c). For rs429358 *(ε4)*, this value exceeded the upper 95th percentile of all single‐site F_ST_ values on chromosome 19 for the population pairs WHG/EF, WHG/TSI, and WHG/IBS and the 97.5th percentile for WHG/LF (Figure 3c,d,e), suggesting that the *ε3/ε4* alleles were likely under selection in the WHG and/or EF. We did not detect any signs of selection for rs7412 (*ε2*) that could have explained the increase in *ε2* seen in northern European populations following the steppe expansion. This was most likely due to sample size constraints combined with the low overall frequency of *ε2*. Although the mean Tajima's D value of the *TOMM40‐APOE‐APOC1* locus did not fall below the 5th or above the 95th percentile thresholds (of all measured Tajima's D values across chromosome 19), the *TOMM40‐APOE‐APOC1* Tajima's D value for EF, LF, and offspring Bronze Age populations was consistently lower than the respective median Tajima's D values across chromosome 19 (Figure 3e). This finding may hint at previous selective sweeps that affected *APOE* in the early farming populations of Europe, possibly explaining the higher *ε3* frequency that we observed in EF compared with WHG.

## 4 DISCUSSION

In this study, we compiled the to‐date largest set of diploid calls for the two most important *APOE* polymorphisms from published Eurasian ancient DNA samples. We found that the *ε4* frequency was significantly higher in WHG than in the first farming populations or modern Europeans. Although the *ε4* allele is considered detrimental to late‐life health, it has also beneficial effects on early‐life fitness today (i.e., antagonistic pleiotropy) (Finch, 2010; Huebbe et al., 2011; van Exel et al., 2017). In addition, it has been shown to be the ancestral allele (Fullerton et al., 2000) and likely benefited our ancestors. Hence, *ε4* probably represented an adaptation to the hunter‐gatherer lifestyle, the only form of subsistence in all human ancestors until the introduction of agriculture. Modern indigenous populations with a long history as hunter‐gatherers show similarly high frequencies of *ε4* as the WHG (Singh et al., 2006) and seem to be exempt from its detrimental effects (Vasunilashorn et al., 2011). The generally heightened inflammatory response associated with *ε4* (Dose et al., 2016; Gale et al., 2014) may have protected the WHG from certain viral and bacterial pathogens, as it has been shown, for example, for malaria and hepatitis C in modern populations (Dose et al., 2016; Fujioka et al., 2014; Wozniak et al., 2002). Additionally, recent research has revealed that *ε4* is associated with improved cognitive development and increased fertility in a high‐pathogen environment (Oriá et al., 2010; van Exel et al., 2017). The *ε4* allele may also have helped to maintain a stable vitamin D metabolism in the dark‐skinned WHG during low sunlight conditions of (especially northern) Europe (Huebbe et al., 2011; Olalde et al., 2014). Although we do not know if *ε4* was already associated with age‐dependent detrimental effects in the WHG, their high levels of aerobic exercise (compared with the average modern European) could have relaxed these constraints (Raichlen & Alexander, 2014). Furthermore, *ε3/ε4*, which is the most common genotype amongst WHG presented in this study, has been significantly associated with increased VO_2max_ (a measure for cardiovascular fitness) after exercise relative to the other *APOE* genotypes (Huebbe et al., 2015). This may have been beneficial to the WHG who routinely went for long periods of endurance running or walking.

In contrast to the WHG, the EF had the lowest *ε4* frequency out of all ancient populations and modern European references. Our F_ST_‐analysis showed that the deviation in frequency of the *ε4*‐SNP (rs429358) between WHG and EF was more extreme than over 95% of all other tested sites on chromosome 19, suggesting selection acted on the *APOE* alleles. The results from our Tajima's D analysis hint at possible selective sweeps in the *APOE* region of EF/LF, which may explain their low *ε4* and high *ε3* frequency, respectively. The transition from hunter‐gathering to farming was one of the most drastic shifts in subsistence in human history. Food became monotonous and supply susceptible to bad harvests, while physical activity shifted from aerobic exercise (endurance running) to more strenuous labour, which negatively affected overall health. There was also an increase of non‐infectious inflammagens from various sources, such as indoor smoke caused by domestic fire, the intake of saturated fats, milk and gluten, and the cooking of food in general (Finch, 2012). This may have resulted in a mismatch between a highly pro‐inflammatory environment (Finch, 2012) and the pro‐inflammatory *ε4* allele (Gale et al., 2014), possibly further increasing the risk for inflammatory disorders such as cardiovascular disease (CVD). While there are signs that CVD was already present in the late Neolithic/Copper Age (Gostner et al., 2011), we cannot say if the *ε4* allele was also associated with CVD in past populations. Furthermore, recent aDNA studies have indicated that infectious agents may have played a lesser role in the Neolithic than previously assumed (Fuchs et al., 2019), relativizing any protective effects of *ε4* against pathogens in this period.

In our data, the observed *ε4* frequency of the LF can be explained by demographic processes, namely the admixture between its parental populations, the WHG and EF. The same can be said for offspring populations of the steppe and LF. Many studies have already described the north‐to‐south cline of *ε4* in modern Europe and attributed it largely to selection (Eisenberg et al., 2010). We have shown here that the higher *ε4* frequency observed in both ancient and modern northern European populations (Bronze Age/Iron Age North, Viking, CEU + GBR) is instead likely the result of a higher steppe ancestral component. It is intriguing that the *ε4* frequencies have largely remained stable for ~4500 years following the last major demographic event in Europe. If we assume that the farming subsistence was the main selective force against the *ε4* allele, we would have expected to see a decrease in its frequency over the past millennia. It is possible that the increase in population density and a concurrently higher infectious burden in later times (Bonczarowska et al., 2022) could have favoured the maintenance of the *ε4* allele.

Compared with any other ancient or modern European population, the EF had the highest *ε3* frequency. We hypothesize that *ε3*‐ vs. *ε4*‐carriers in early Neolithic populations were more resistant to famines (i.e., those caused by a bad harvest). This is in line with findings that show that the *ε3* allele is associated with the potential to more efficiently use dietary energy and deposit fat in adipose tissue (Huebbe et al., 2015).

Interestingly, we did not detect *ε2* in either the WHG or a smaller population of eastern hunter‐gatherers (EHG; *N* = 13, Table S2), suggesting that *ε2* may have been completely lost or reduced to negligible frequencies in the hunter‐gatherer populations of Mesolithic Europe. Astonishingly, the same observation can be made for many modern indigenous hunter‐gatherers from South and Central America (Marin et al., 1997; Vasunilashorn et al., 2011) or for Australian aborigines (Kamboh et al., 1991). Although it is possible that this decrease in allele frequency (or rather allele loss) happened as a result of genetic drift, it is unlikely that both WHG and EHG as well as modern hunter‐gatherers were independently affected in the same manner. Rather, it seems that the evolutionarily older *ε3* and *ε4* alleles (Fullerton et al., 2000) were more advantageous for WHG.

Based on the results from this study, it seems that the *ε2* allele was (re)introduced to the European gene pool after the arrival of the EF (and later the steppe populations). Recent research has shown an association of the murine gut microbiome with *APOE* status. Mice expressing the human APOE2 protein had increased relative abundance of the order *Clostridiales* and family *Ruminococcaceae* (Tran et al., 2019). This may have been advantageous in adapting to the Neolithic diet. For instance, repeated heating and cooling of starch‐rich foods increase the content of resistant starches, and it has been shown that especially *Ruminococcaceae* may help with their digestion (Umu et al., 2015). It is plausible that the EF handled food in this manner and that their diet had a higher overall starch content compared with WHG (Ollivier et al., 2016).

Following the two major demographic events, the observed *ε2* frequency did not significantly differentiate from the admixture‐informed expected frequency in populations before the common era. However, the observed *ε2* frequency was significantly higher than its expected frequency in all common era populations (i.e., Viking, IBS + TSI, CEU + GBR). This was especially noticeable in northern Europeans, where *ε2* frequencies were higher than in any ancestral population, suggesting that admixture alone was not responsible for this increase. However, the overall increase was rather modest, which may explain why we did not detect any signatures of selection.

We conclude that the *APOE* allele frequencies of modern Europeans, which strongly influence the likelihood of becoming long‐lived today, are most probably the consequence of the past demographic processes and adaptations outlined in this study. The different *APOE* allele frequencies of the WHG and EF likely resulted from adaptations to diet, physical activity and inflammatory load (Finch, 2012). We have also shown that the north‐to‐south cline of *ε4* in modern Europe was the result of a higher steppe admixture rather than of selection/environmental differences. The persistence of the *ε4* allele since the Bronze Age may be attributed to a rise in infectious diseases. It remains uncertain whether the increase in *ε2* frequency in modern populations was the result of selection and, if so, which selective pressures may have been involved. Furthermore, it is unclear if and to what extent the *APOE* alleles already influenced longevity in past populations. Future association studies using ancient samples would be needed, which in turn would require accurate anthropological documentation of age at death. Longevity is a post‐reproductive trait and therefore unlikely to have been the main driver of selection, unless we assume that a grandmother/grandfather effect played a positive role in past populations.

## 5 LIMITATIONS

Unfortunately, the overall quality of aDNA data is much lower than of modern genomic data. While we made sure to use only higher quality data for our analyses, the number of samples that passed this quality control was limited, which in turn restricted statistical power.

## AUTHOR CONTRIBUTIONS

Idea/concept: AN and BKK. Data curation/processing: DK and NdS. Data analysis: DK. Interpretation: DK, AN, and BKK. Writing: DK. Advice: AC, GT, and JD.

## CONFLICT OF INTEREST STATEMENT

The authors declare no competing interests.

## Supporting information


Data S1:

## Data Availability

Data sharing is not applicable to this article as no new data were created or analyzed in this study.

## References

[acel13819-bib-0047] Abondio, P. , Sazzini, M. , Garagnani, P. , Boattini, A. , Monti, D. , Franceschi, C. , Luiselli, D. , & Giuliani, C. (2019). The genetic variability of APOE in different human populations and Its implications for longevity. Genes, 10, 222. 10.3390/genes10030222 30884759 PMC6471373

[acel13819-bib-0002] Alexander, D. H. , Novembre, J. , & Lange, K. (2009). Fast model‐based estimation of ancestry in unrelated individuals. Genome Research, 19, 1655–1664. 10.1101/gr.094052.109 19648217 PMC2752134

[acel13819-bib-0003] Benjamini, Y. , & Hochberg, Y. (1995). Controlling the false discovery rate: A practical and powerful approach to multiple testing. Journal of the Royal Statistical Society: Series B (Methodological), 57, 289–300. 10.1111/j.2517-6161.1995.tb02031.x

[acel13819-bib-0048] Bonczarowska, J. H. , Susat, J. , Mühlemann, B. , Jasch‐Boley, I. , Brather, S. , Höke, B. , Brather‐Walter, S. , Schoenenberg, V. , Scheschkewitz, J. , Graenert, G. , Krausse, D. , Francken, M. , Jones, T. C. , Wahl, J. , Nebel, A. , & Krause‐Kyora, B. (2022). Pathogen genomics study of an early medieval community in Germany reveals extensive co‐infections. Genome Biology, 23, 250. 10.1186/s13059-022-02806-8 36510283 PMC9746117

[acel13819-bib-0005] Cruts, M. , & van Broeckhoven, C. (1998). Molecular genetics of Alzheimer's disease. Annals of Medicine, 30, 560–565. 10.3109/07853899809002605 9920359

[acel13819-bib-0046] Danecek, P. , Auton, A. , Abecasis, G. , Albers, C. A. , Banks, E. , DePristo, M. A. , Handsaker, R. E. , Lunter, G. , Marth, G. T. , Sherry, S. T. , McVean, G. , Durbin, R. , & 1000 Genomes Project Analysis Group . (2011). The variant call format and VCFtools. Bioinformatics, 27, 2156–2158. 10.1093/bioinformatics/btr330 21653522 PMC3137218

[acel13819-bib-0049] Deelen, J. , Evans, D. S. , Arking, D. E. , Tesi, N. , Nygaard, M. , Liu, X. , Wojczynski, M. K. , Biggs, M. L. , van der Spek, A. , Atzmon, G. , Ware, E. B. , Sarnowski, C. , Smith, A. V. , Seppälä, I. , Cordell, H. J. , Dose, J. , Amin, N. , Arnold, A. M. , Ayers, K. L. , … Murabito, J. M. (2019). A meta‐analysis of genome‐wide association studies identifies multiple longevity genes. Nature Communications, 10, 3669. 10.1038/s41467-019-11558-2 PMC6694136 31413261

[acel13819-bib-0008] Dose, J. , Huebbe, P. , Nebel, A. , & Rimbach, G. (2016). APOE genotype and stress response—A mini review. Lipids in Health and Disease, 15, 121. 10.1186/s12944-016-0288-2 27457486 PMC4960866

[acel13819-bib-0009] Eisenberg, D. T. A. , Kuzawa, C. W. , & Hayes, M. G. (2010). Worldwide allele frequencies of the human apolipoprotein E gene: Climate, local adaptations, and evolutionary history. American Journal of Physical Anthropology, 143, 100–111. 10.1002/ajpa.21298 20734437

[acel13819-bib-0010] Finch, C. (2012). Evolution of the human lifespan, past, present, and future: Phases in the evolution of human life expectancy in relation to the inflammatory load. Proceedings of the American Philosophical Society, 156, 9–44. 23035388

[acel13819-bib-0011] Finch, C. E. (2010). Evolution of the human lifespan and diseases of aging: Roles of infection, inflammation, and nutrition. Proceedings of the National Academy of Sciences, 107, 1718–1724. 10.1073/pnas.0909606106 PMC2868286 19966301

[acel13819-bib-0012] Fuchs, K. , Rinne, C. , Drummer, C. , Immel, A. , Krause‐Kyora, B. , & Nebel, A. (2019). Infectious diseases and Neolithic transformations: Evaluating biological and archaeological proxies in the German loess zone between 5500 and 2500 BCE. The Holocene, 29, 1545–1557. 10.1177/0959683619857230

[acel13819-bib-0013] Fujioka, H. , Phelix, C. F. , Friedland, R. P. , Zhu, X. , Perry, E. A. , Castellani, R. J. , & Perry, G. (2014). Apolipoprotein E4 prevents growth of malaria at the intraerythrocyte stage: Implications for differences in racial susceptibility to Alzheimer's disease. Journal of Health Care for the Poor and Underserved, 24, 70–78. 10.1353/hpu.2014.0009 PMC4909051 24241262

[acel13819-bib-0050] Fullerton, S. M. , Clark, A. G. , Weiss, K. M. , Nickerson, D. A. , Taylor, S. L. , Stengård, J. H. , Salomaa, V. , Vartiainen, E. , Perola, M. , Boerwinkle, E. , & Sing, C. F. (2000). Apolipoprotein E variation at the sequence haplotype level: Implications for the origin and maintenance of a major human polymorphism. The American Journal of Human Genetics, 67, 881–900. 10.1086/303070 10986041 PMC1287893

[acel13819-bib-0051] Gale, S. C. , Gao, L. , Mikacenic, C. , Coyle, S. M. , Rafaels, N. , Murray Dudenkov, T. , Madenspacher, J. H. , Draper, D. W. , Ge, W. , Aloor, J. J. , Azzam, K. M. , Lai, L. , Blackshear, P. J. , Calvano, S. E. , Barnes, K. C. , Lowry, S. F. , Corbett, S. , Wurfel, M. M. , & Fessler, M. B. (2014). APOε4 is associated with enhanced in vivo innate immune responses in human subjects. Journal of Allergy and Clinical Immunology, 134, 127–134.e9. 10.1016/j.jaci.2014.01.032 24655576 PMC4125509

[acel13819-bib-0016] Gostner, P. , Pernter, P. , Bonatti, G. , Graefen, A. , & Zink, A. R. (2011). New radiological insights into the life and death of the Tyrolean iceman. Journal of Archaeological Science, 38, 3425–3431. 10.1016/j.jas.2011.08.003

[acel13819-bib-0052] Haak, W. , Lazaridis, I. , Patterson, N. , Rohland, N. , Mallick, S. , Llamas, B. , Brandt, G. , Nordenfelt, S. , Harney, E. , Stewardson, K. , Fu, Q. , Mittnik, A. , Bánffy, E. , Economou, C. , Francken, M. , Friederich, S. , Pena, R. G. , Hallgren, F. , Khartanovich, V. , … Reich, D. (2015). Massive migration from the steppe was a source for Indo‐European languages in Europe. Nature, 522, 207–211. 10.1038/nature14317 25731166 PMC5048219

[acel13819-bib-0053] Huebbe, P. , Dose, J. , Schloesser, A. , Campbell, G. , Glüer, C.‐C. , Gupta, Y. , Ibrahim, S. , Minihane, A.‐M. , Baines, J. F. , Nebel, A. , & Rimbach, G. (2015). Apolipoprotein E (APOE) genotype regulates body weight and fatty acid utilization—Studies in gene‐targeted replacement mice. Molecular Nutrition & Food Research, 59, 334–343. 10.1002/mnfr.201400636 25381750

[acel13819-bib-0054] Huebbe, P. , Nebel, A. , Siegert, S. , Moehring, J. , Boesch‐Saadatmandi, C. , Most, E. , Pallauf, J. , Egert, S. , Müller, M. J. , Schreiber, S. , Nöthlings, U. , & Rimbach, G. (2011). APOE ε4 is associated with higher vitamin D levels in targeted replacement mice and humans. The FASEB Journal, 25, 3262–3270. 10.1096/fj.11-180935 21659554

[acel13819-bib-0055] Immel, A. , Pierini, F. , Rinne, C. , Meadows, J. , Barquera, R. , Szolek, A. , Susat, J. , Böhme, L. , Dose, J. , Bonczarowska, J. , Drummer, C. , Fuchs, K. , Ellinghaus, D. , Kässens, J. C. , Furholt, M. , Kohlbacher, O. , Schade‐Lindig, S. , Franke, A. , Schreiber, S. , … Krause‐Kyora, B. (2021). Genome‐wide study of a Neolithic Wartberg grave community reveals distinct HLA variation and hunter‐gatherer ancestry. Communications Biology, 4, 113. 10.1038/s42003-020-01627-4 33495542 PMC7835224

[acel13819-bib-0021] Kamboh, M. , Serjeantson, S. , & Ferrell, R. (1991). Genetic studies of human apolipoproteins. XVIII. Apolipoprotein polymorphisms in Australian aborigines. Human Biology, 63, 179–186. 2019410

[acel13819-bib-0022] Li, H. (2011). A statistical framework for SNP calling, mutation discovery, association mapping and population genetical parameter estimation from sequencing data. Bioinformatics, 27, 2987–2993. 10.1093/bioinformatics/btr509 21903627 PMC3198575

[acel13819-bib-0056] Li, H. , Handsaker, B. , Wysoker, A. , Fennell, T. , Ruan, J. , Homer, N. , Marth, G. , Abecasis, G. , Durbin, R. , & 1000 Genome Project Data Processing Subgroup . (2009). The sequence alignment/map format and SAMtools. Bioinformatics, 25, 2078–2079. 10.1093/bioinformatics/btp352 19505943 PMC2723002

[acel13819-bib-0024] Lucotte, G. , Loirat, F. , & Hazout, S. (1997). Pattern of gradient of apolipoprotein E allele *4 frequencies in western Europe. Human Biology, 69, 253–262. 9057348

[acel13819-bib-0025] Mahley, R. W. (2016). Apolipoprotein E: From cardiovascular disease to neurodegenerative disorders. Journal of Molecular Medicine, 94, 739–746. 10.1007/s00109-016-1427-y 27277824 PMC4921111

[acel13819-bib-0026] Marciniak, S. , & Perry, G. H. (2017). Harnessing ancient genomes to study the history of human adaptation. Nature Reviews Genetics, 18, 659–674. 10.1038/nrg.2017.65 28890534

[acel13819-bib-0027] Marin, G. , Tavella, M. , Guerreiro, J. , Santos, S. , & Zago, M. (1997). Absence of the E2 allele of apolipoprotein in Amerindians. Brazilian Journal of Genetics, 20, 741–743.

[acel13819-bib-0057] Mathieson, I. , Lazaridis, I. , Rohland, N. , Mallick, S. , Patterson, N. , Roodenberg, S. A. , Harney, E. , Stewardson, K. , Fernandes, D. , Novak, M. , Sirak, K. , Gamba, C. , Jones, E. R. , Llamas, B. , Dryomov, S. , Pickrell, J. , Arsuaga, J. L. , de Castro, J. M. B. , Carbonell, E. , … Reich, D. (2015). Genome‐wide patterns of selection in 230 ancient Eurasians. Nature, 528, 499–503. 10.1038/nature16152 26595274 PMC4918750

[acel13819-bib-0058] Mölder, F. , Jablonski, K. P. , Letcher, B. , Hall, M. B. , Tomkins‐Tinch, C. H. , Sochat, V. , Forster, J. , Lee, S. , Twardziok, S. O. , Kanitz, A. , Wilm, A. , Holtgrewe, M. , Rahmann, S. , Nahnsen, S. , & Köster, J. (2021). Sustainable data analysis with Snakemake. F1000Research, 10, 33. 10.12688/f1000research.29032.1 34035898 PMC8114187

[acel13819-bib-0059] Nebel, A. , Kleindorp, R. , Caliebe, A. , Nothnagel, M. , Blanché, H. , Junge, O. , Wittig, M. , Ellinghaus, D. , Flachsbart, F. , Wichmann, H.‐E. , Meitinger, T. , Nikolaus, S. , Franke, A. , Krawczak, M. , Lathrop, M. , & Schreiber, S. (2011). A genome‐wide association study confirms APOE as the major gene influencing survival in long‐lived individuals. Mechanisms of Ageing and Development, 132, 324–330. 10.1016/j.mad.2011.06.008 21740922

[acel13819-bib-0060] Olalde, I. , Allentoft, M. E. , Sánchez‐Quinto, F. , Santpere, G. , Chiang, C. W. K. , DeGiorgio, M. , Prado‐Martinez, J. , Rodríguez, J. A. , Rasmussen, S. , Quilez, J. , Ramírez, O. , Marigorta, U. M. , Fernández‐Callejo, M. , Prada, M. E. , Encinas, J. M. V. , Nielsen, R. , Netea, M. G. , Novembre, J. , Sturm, R. A. , … Lalueza‐Fox, C. (2014). Derived immune and ancestral pigmentation alleles in a 7,000‐year‐old Mesolithic European. Nature, 507, 225–228. 10.1038/nature12960 24463515 PMC4269527

[acel13819-bib-0061] Ollivier, M. , Tresset, A. , Bastian, F. , Lagoutte, L. , Axelsson, E. , Arendt, M.‐L. , Bălăşescu, A. , Marshour, M. , Sablin, M. V. , Salanova, L. , Vigne, J.‐D. , Hitte, C. , & Hänni, C. (2016). Amy2B copy number variation reveals starch diet adaptations in ancient European dogs. Royal Society Open Science, 3, 160449 10.1098/rsos.160449 28018628 PMC5180126

[acel13819-bib-0062] Oriá, R. B. , Patrick, P. D. , Oriá, M. O. B. , Lorntz, B. , Thompson, M. R. , Azevedo, O. G. R. , Lobo, R. N. B. , Pinkerton, R. F. , Guerrant, R. L. , & Lima, A. A. M. (2010). ApoE polymorphisms and diarrheal outcomes in Brazilian shanty town children. Brazilian Journal of Medical and Biological Research, 43, 249–256. 10.1590/S0100-879X2010007500003 20401432 PMC3057459

[acel13819-bib-0034] Plotly Technologies Inc . (2015). Collaborative data science Publisher. Plotly Technologies Inc. Retrieved from https://plot.ly

[acel13819-bib-0035] Raichlen, D. A. , & Alexander, G. E. (2014). Exercise, APOE genotype, and the evolution of the human lifespan. Trends in Neurosciences, 37, 247–255. 10.1016/j.tins.2014.03.001 24690272 PMC4066890

[acel13819-bib-0063] Shinohara, M. , Kanekiyo, T. , Tachibana, M. , Kurti, A. , Shinohara, M. , Fu, Y. , Zhao, J. , Han, X. , Sullivan, P. M. , Rebeck, G. W. , Fryer, J. D. , Heckman, M. G. , & Bu, G. (2020). APOE2 is associated with longevity independent of Alzheimer's disease. eLife, 9, e62199. 10.7554/eLife.62199 33074098 PMC7588231

[acel13819-bib-0037] Singh, P. P. , Singh, M. , & Mastana, S. S. (2006). APOE distribution in world populations with new data from India and the UK. Annals of Human Biology, 33, 279–308. 10.1080/03014460600594513 17092867

[acel13819-bib-0064] The 1000 Genomes Project Consortium , Auton, A. , Abecasis, G. R. , Steering committee , Altshuler, D. M. , Durbin, R. M. , Abecasis, G. R. , Bentley, D. R. , Chakravarti, A. , Clark, A. G. , Donnelly, P. , Eichler, E. E. , Flicek, P. , Gabriel, S. B. , Gibbs, R. A. , Green, E. D. , Hurles, M. E. , Knoppers, B. M. , Korbel, J. O. , … Abecasis, G. R. (2015). A global reference for human genetic variation. Nature, 526, 68–74. 10.1038/nature15393 26432245 PMC4750478

[acel13819-bib-0065] Tran, T. T. T. , Corsini, S. , Kellingray, L. , Hegarty, C. , Le Gall, G. , Narbad, A. , Müller, M. , Tejera, N. , O’Toole, P. W. , Minihane, A.‐M. , & Vauzour, D. (2019). APOE genotype influences the gut microbiome structure and function in humans and mice: Relevance for Alzheimer's disease pathophysiology. The FASEB Journal, 33, 8221–8231. 10.1096/fj.201900071R 30958695 PMC6593891

[acel13819-bib-0066] Umu, Ö. C. O. , Frank, J. A. , Fangel, J. U. , Oostindjer, M. , da Silva, C. S. , Bolhuis, E. J. , Bosch, G. , Willats, W. G. T. , Pope, P. B. , & Diep, D. B. (2015). Resistant starch diet induces change in the swine microbiome and a predominance of beneficial bacterial populations. Microbiome, 3, 16. 10.1186/s40168-015-0078-5 25905018 PMC4405844

[acel13819-bib-0067] van Exel, E. , Koopman, J. J. E. , Bodegom, D. v. , Meij, J. J. , Knijff, P. d. , Ziem, J. B. , Finch, C. E. , & Westendorp, R. G. J. (2017). Effect of APOE ε4 allele on survival and fertility in an adverse environment. PLoS One, 12, e0179497. 10.1371/journal.pone.0179497 28683096 PMC5500260

[acel13819-bib-0042] Van Rossum, G. , & Drake, F. (2009). Python 3 reference manual. CreateSpace.

[acel13819-bib-0068] Vasunilashorn, S. , Finch, C. E. , Crimmins, E. M. , Vikman, S. A. , Stieglitz, J. , Gurven, M. , Kaplan, H. , & Allayee, H. (2011). Inflammatory gene variants in the Tsimane, an indigenous Bolivian population with a high infectious load. Biodemography and Social Biology, 57, 33–52. 10.1080/19485565.2011.564475 21845926 PMC3529658

[acel13819-bib-0069] Virtanen, P. , Gommers, R. , Oliphant, T. E. , Haberland, M. , Reddy, T. , Cournapeau, D. , Burovski, E. , Peterson, P. , Weckesser, W. , Bright, J. , van der Walt, S. J. , Brett, M. , Wilson, J. , Millman, K. J. , Mayorov, N. , Nelson, A. R. J. , Jones, E. , Kern, R. , Larson, E. , … Vázquez‐Baeza, Y. (2020). SciPy 1.0: Fundamental algorithms for scientific computing in Python. Nature Methods, 17, 261–272. 10.1038/s41592-019-0686-2 32015543 PMC7056644

[acel13819-bib-0045] Wozniak, M. A. , Itzhaki, R. F. , Faragher, E. B. , James, M. W. , Ryder, S. D. , & Irving, W. L. (2002). Apolipoprotein E‐ϵ4 protects against severe liver disease caused by hepatitis C virus. Hepatology, 36, 456–463. 10.1053/jhep.2002.34745 12143056

